# Local MRI before and after Tumor Resection in Neuroblastoma: Impact of Residual Disease on Event Free Survival

**DOI:** 10.3390/jcm12237297

**Published:** 2023-11-24

**Authors:** Jürgen F. Schäfer, Sebastian Gassenmaier, Steven Warmann, Cristian Urla, Leonie Frauenfeld, Tim Flaadt, Maryanna Chaika, Michael Esser, Ilias Tsiflikas, Beate Timmermann, Jörg Fuchs

**Affiliations:** 1 Division of Pediatric Radiology, Department of Radiology, University Hospital Tübingen, 72076 Tübingen, Germany; sebastian.gassenmaier@med.uni-tuebingen.de (S.G.); maryanna.chaika@med.uni-tuebingen.de (M.C.); michael.esser@med.uni-tuebingen.de (M.E.);; 2 Department of Pediatric Surgery and Pediatric Urology, University Hospital Tübingen, 72076 Tübingen, Germany; steven.warmann@med.uni-tuebingen.de (S.W.); cristian.urla@med.uni-tuebingen.de (C.U.); joerg.fuchs@med.uni-tuebingen.de (J.F.); 3 Department of Pathology, University Hospital Tübingen, 72076 Tübingen, Germany; leonie.frauenfeld@med.uni-tuebingen.de; 4 Department of Pediatric Oncology and Hematology, University Hospital Tübingen, 72076 Tübingen, Germany; tim.flaadt@med.uni-tuebingen.de; 5 Particle Therapy Clinic and the West German Proton Therapy Centre, University Hospital Essen, 45147 Essen, Germany; beate.timmermann@uk-essen.de

**Keywords:** high-risk neuroblastoma, residual tumor, MRI, surgical resection, EFS, irradiation

## Abstract

(1) Background: The study aimed to investigate the influence of MRI-defined residual disease on local tumor control after resection of neuroblastic tumors in patients without routine adjuvant radiotherapy. (2) Methods: Patients, who underwent tumor resection between 2009 and 2019 and received a pre- and postoperative MRI, were included in this retrospective single-center study. Measurement of residual disease (RD) was performed using standardized criteria. Primary endpoint was the local or combined (local and metastatic) event free survival (EFS). (3) Results: Forty-one patients (20 female) with median age of 39 months were analyzed. Risk group analysis showed eleven low-, eight intermediate-, and twenty-two high-risk patients (LR, IR, HR). RD was found in 16 cases by MRI. A local or combined relapse or progression was found in nine patients of whom eight patients had RD (*p* = 0.0004). From the six patients with local or combined relapse in the HR group, five had RD (*p* = 0.005). Only one of 25 patients without RD had a local event. Mean EFS (month) was significantly higher if MRI showed no residual tumor (81 ± 5 vs. 43 ± 9; *p* = 0.0014) for the total cohort and the HR subgroup (62 ± 7 vs. 31 ± 11; *p* = 0.016). (4) Conclusions: In our series, evidence of residual tumor, detectable by MRI, was associated with insufficient local control, resulting in relapses or local progression in 50% of patients. Only one of the patients without residual tumor had a local relapse.

## 1. Introduction

Neuroblastoma is the most common extracranial solid tumor in pediatric patients. A particular challenge is the pronounced biological heterogeneity of neuroblastoma with completely different treatment options ranging from observation to stem cell therapy [1,2,3]. Common imaging modalities for diagnosis and therapy response assessment as well as follow-up include morphological imaging such as ultrasound, computed tomography (CT), and magnetic resonance imaging (MRI) [4,5]. Recent developments and technical progression strengthened the clinical importance of functional imaging such as positron-emission tomography (PET) or diffusion-weighted imaging (DWI) [6,7]. A further standard imaging procedure comprises 123iodine-meta-iodobenzylguanidine (123ImIBG) scintigraphy [4,8]. These imaging methods play a pivotal role in staging of neuroblastic tumors using image-defined risk factors (IDRFs) of the International Neuroblastoma Risk Group Staging System (INRGSS) [5,9,10,11]. Risk stratification is based on age, INSS stage, and N-MYC amplification in most studies [12,13]. In the latest revision of the Children’s Oncology Group (COG), the INRGSS and segmental chromosome aberrations (SCAs) are also taken into consideration [14]. Therapy includes systemic therapy with chemotherapy and immunotherapy, and local therapy with surgery resection and irradiation [15,16,17,18]. Although irradiation of the tumor bed (with or without boost) is currently recommended in high-risk as well as in intermediate-risk (plus residual disease) patients, according to current guidelines, this procedure is discussed controversially [19]. In this therapeutic approach, patients receive 21 Gy on the preoperative tumor volume and a boost of 14.4 Gy on the macroscopic residual tumor volume. While previous reports favored local radiotherapy in order to reduce local failure rate [20], recent data suggest that therapy de-escalation might be possible in patients with more than 90% tumor resection and no primary IDRF [21]. Furthermore, recent data from the COG ANBL0532 trial and Holmes et al. demonstrated that the risk of local progression rate was not reduced by boost radiation therapy to gross residual disease [22] and progression and relapse rate is associated with macroscopic incomplete resection [23]. This is especially relevant as the reduction or omission of local radiation therapy reduces the therapy-associated toxicity in these very young patients [24].

However, the major issue demonstrates the lack of standardization for determination of complete resection including a reliable quantitative measurement with objective criteria instead of using surgical reports only. Therefore, the purpose of this retrospective study was to analyze the occurrence of tumor progression in dependence of residual tumor volume detected by MRI in a cohort that did not receive adjuvant radiotherapy.

## 2. Materials and Methods

**Figure 1:**
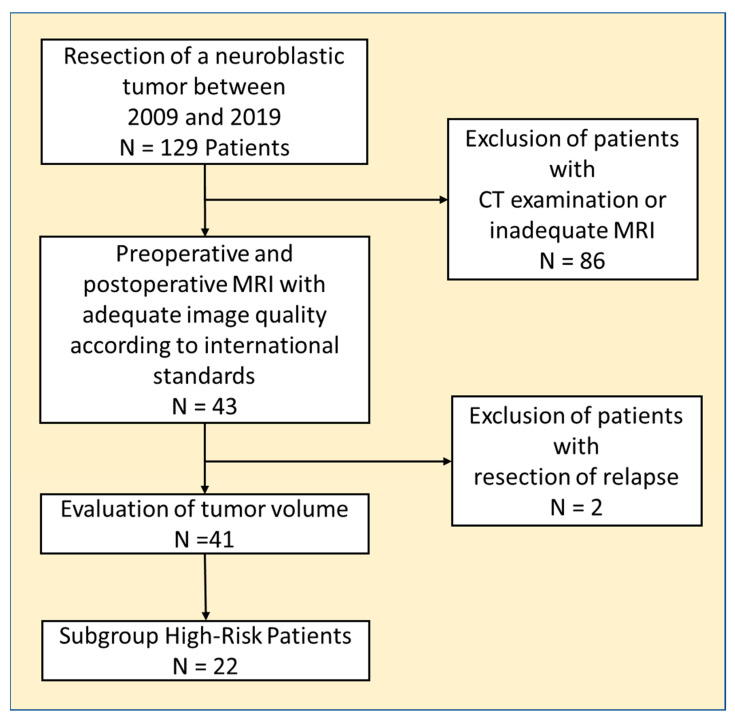
The graph shows the workflow of patient selection and the evaluation of the entire cohort as well as the subgroup of high-risk patients.

The local institutional review board approved this retrospective, monocentric study (481/2015BO2). All pediatric patients referred to our national reference center for resection of neuroblastic tumors between 2009 and 2019 were reviewed. All patients were treated according to the current national and international trials or therapy recommendations. Characteristically, treatment of high-risk neuroblastoma was performed according to the GPOH NB2004 high-risk trial protocol or NB2016 registry protocol. Tumor bed irradiation to the extent of the preoperative tumor margins was not provided here. Patients did not receive postoperative tumor bed radiation. The inclusion criterion was the presence of a pre- and postoperative MRI of the primary tumor region. The quality of imaging had to meet the generally accepted standards [5]. Patients were grouped according to their clinical data into low-risk (LR), intermediate-risk (IR), and high-risk patients (HR) (Figure 1). Risk grouping, tumor progression events, and overall survival were analyzed using the institutional databank and data from clinical studies, including registry databases of the GPOH (German society of pediatric hematology and oncology).

**Figure 2:**
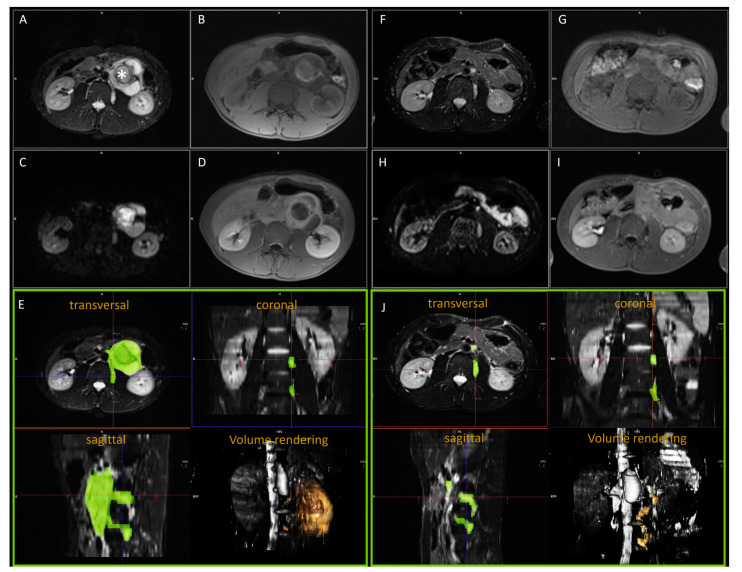
Example of imaging analysis in a 15-year-old male patient with ganglioneuroblastoma. (**A**–**E**) Before and (**F**–**J**) after resection. (**A**,**F**) T2 weighted images with fat saturation. (**B**,**G**) Non-enhanced T1 weighted images and (**D**,**I**) after application of contrast medium. (**C**,**H**) DWI with high b-value (ADC map not shown). (**E**,**J**) The multiplanar reconstruction respective 3D volume analysis; the tumor volume is colored green, and in the volume rendering method it is colored light yellow. Before resection, the tumor located anterior to the spine reached the left renal hilum and had extensions into the ipsilateral neuroforamina. MRI characterized the tumor as inhomogeneous with hemorrhage (star), inhomogeneous to even absent contrast enhancement. There was a moderate diffusion restriction in the area of the non-regressively altered tumor parts. Tumor volume was 234 mL. After resection, two extensions could be detected unchanged in the neuroforamina and a small RD adjacent to the aorta. The total volume was 11 mL. According to the surgical report, the extensions into the neuroforamina were not resected (IME) because this patient did not have a high-risk situation. There was no tumor progression.

The MRI protocol usually comrpised T2 weighted sequences with fat saturation, diffusion-weighted sequences (DWI) with at least two b-values to calculate the apparent diffusion coefficient (ADC), and T1 weighted sequences before and after the application of contrast medium. Postoperative resection status was evaluated by a senior pediatric radiologist with more than 25 years of experience using pre- and postoperative MRI (Figure 2). A residual tumor was defined as conspicuous tissue with typical MRI characteristics for neuroblastoma on postoperative imaging, which was located at the same site and with comparable signaling on the preoperative imaging. A radiology resident manually segmented all suspicious findings for a residual tumor that were verified by the board-certified pediatric radiologist using a dedicated workstation with standard postprocessing software (syngo, via; vb60a hf30; Siemens Healthineers, Erlangen, Germany) (Figure 2). Both readers were blinded to the surgery report and the clinical outcome. T2 weighted imaging was used for segmentation if available. In blurred images or images hampered by artifacts, T1 weighted post-contrast sequences were used instead. The same imaging sequence was used for preoperative as well as postoperative volumetry [25].

The reference standard was determined by evaluating the surgical report, available follow-up examinations and data, including available MIBG scans, and a final review by a senior pediatric radiologist and a senior pediatric surgeon. The result of the resection state was divided into complete macroscopic excision (CME) and incomplete macroscopic excision (IME). CME was retrospectively defined as a condition where all visible and palpable parts of the primary tumor and regional lymph nodes were removed. All other descriptions (e.g., almost complete or more than 95% tumor removal) were defined as IME. Due to the retrospective study design with blinded evaluation of the MRI examinations, the residual tumors detected by MRI were not usually confirmed histologically; the clinical and imaging follow-up had to support the state and the disease’s progression. The latter was additionally confirmed by histologic evaluation if available. Thus, in the case of local relapse, only these findings were finally rated as true positive even if the origin of the local progression was evident from this location finding.

Statistical analysis was performed using JMP14 (SAS Institute, Cary, NC, USA) and MedCalc version 18.1 (MedCalc Software, Ostend, Belgium). Continuous variables and non-parametric data are displayed as median and interquartile range (IQR) because the Shapiro–Wilk test showed no normal distribution. Subsequently, the Wilcoxon rank sum test and the Chi square test were applied. A subgroup analysis of the high-risk patients was performed. Kaplan–Meier analysis for event free survival time (EFS) and overall survival (OS) after resection was performed. Events were grouped into local and/or metastatic events. Local events were defined as a new tumor in the location of the primary tumor or progression of a residual tumor by 20% or more of the longest diameter according to the International Neuroblastoma Response Criteria [26]. Statistical significance was assumed for *p* < 0.05.

## 3. Results

Of the 127 patients with a neuroblastic tumor operated on between 2009 and 2019, 41 (20 female) patients fulfilled the inclusion criteria (Figure 1). The median age at the time of tumor resection was 39 months (24–71 months). Histopathological diagnosis was NB in twenty-three cases, ganglioneuroblastoma (GNB) in fifteen cases, and ganglioneuroma (GN) in three cases. Preoperative MRI examinations were performed at a median of 12 days (5–30 days) before surgery, and patients received MRI a median of 95 days (45–141 days) after surgery. Risk group stratification resulted in twenty-two high-risk, eight intermediate-risk, and eleven low-risk patients.

The median preoperative tumor volume was 28.1 mL (8.5–76.6 mL). A residual tumor was suspected in 17 patients on postoperative MRI. According to the reference standard, one patient was evaluated as a false positive. Thus, sixteen patients with residual tumors were further analyzed with a median tumor volume of 2.2 mL (0.9–6.4 mL), including eight high-risk patients, two intermediate-risk cases, and six low-risk cases. Assuming a spherical shape, this corresponds to a one-dimensional diameter of about 1.6 cm. Further characteristics are listed in Table 1. CME was reported in 30 cases but confirmed only 23 times by MRI. IME was reported in eleven patients and confirmed nine times by MRI. Thus, the agreement between surgical report and MRI after resection was approximately 78% (32/41).

The mean overall survival was 103 months (±6 SE; 92 to 114 CI 95%). Three patients died during the observation period which were all from the HR group. Two of them were found to have residual tumor on MRI.

**Figure 3:**
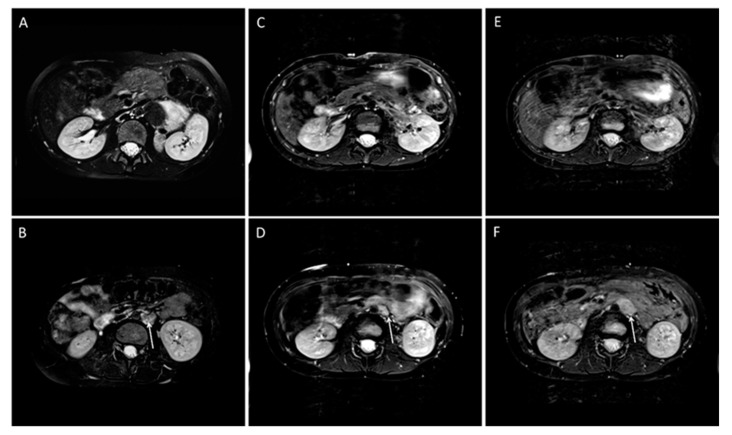
Example of a case with residual tumor and local progression in a 10-year-old girl with ganglioneuroblastoma, N-MYC non-amplified tumor, INSS Stage IV. The T2 weighted images are shown. (**A**,**B**) Before resection, (**C**,**D**) after resection, and E and F during follow-up. (**A**,**C**,**E**) Transversal images at the level of the primary tumor on the left suprarenal side. After surgery and in the follow-up, no tumor can be detected (**C**,**E**). Enlarged left lymph node before surgery (**B**) and after surgery (**D**) (arrows). According to the surgical report, a CME was performed. In the course of 12 months, the tumor progressed (**F**) (arrow).

The mean event free survival was 52 months (±6 SE; 50 to 74 CI 95%). The EFS for local or combined relapse was 66 months (±6 SE; 54 to 77 CI 95%). An event occurred in eleven patients (eight HR, two IR, and one LR), including three local events, six combined (local and metastatic) events, and two metastatic events. These two patients with new metastases had no residual tumors (Table 2). Of the nine cases with local or combined events, a residual tumor was found in eight patients by MRI, whereas the respective surgical report mentioned incomplete resection in three of these cases (Table 2; example in Figure 3). A statistically significant association between residual tumor and the local or combined event was found (n = 41; *p* = 0.0004). There was also a moderate correlation between the number of IDRFs and local EFS (*p* = 0.04). All other influencing factors examined were not significantly different (Table 3).

When the high-risk group was considered separately, six patients had a local or combined event. Five of these patients had residual tumor. A statistically significant association between residual tumor and the local or combined event was found (n = 22; *p* = 0.005). In this subgroup, there were no other factors examined that indicated a statistically significant association. This was also true for the number of IDRFs.

**Figure 4:**
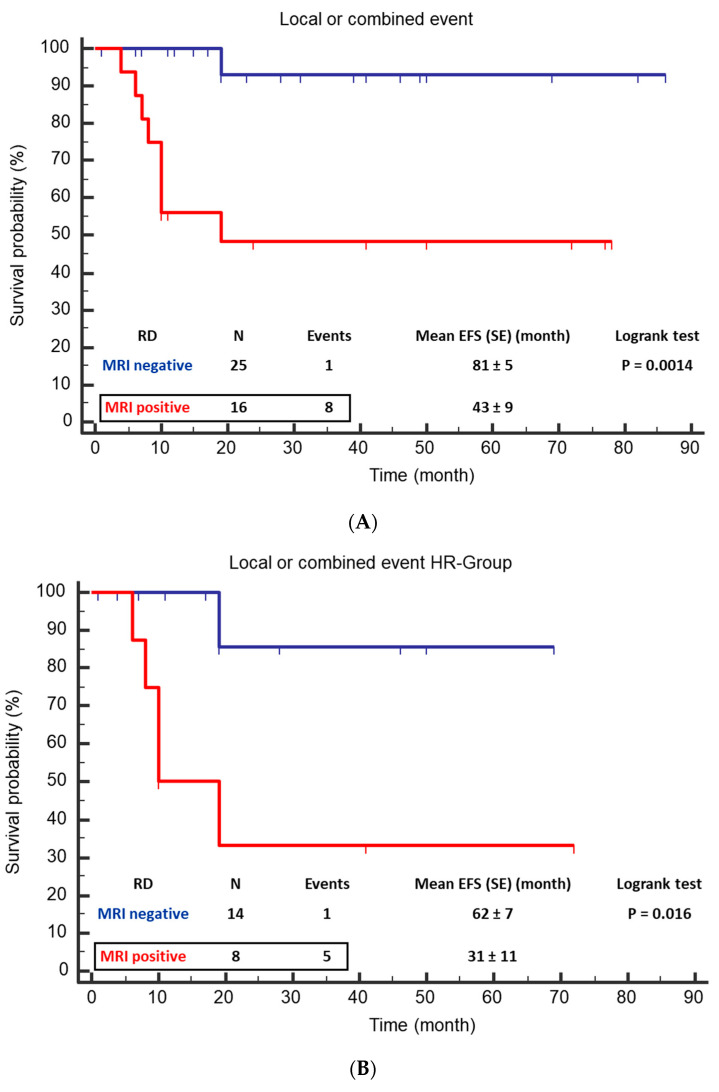
Event free survival (EFS) of patients with positive MRI for residual disease vs. negative MRI. (**A**) For the total cohort, mean EFS was 46 months (CI 95% 30 to 61) for positive MRI and 79 months (CI 95% 70 to 88; *p* = 0.0035) for negative MRI. (**B**) In the HR group, mean EFS was 36 months (CI 95% 16 to 56) vs. 63 months (CI 95% 51 to 75).

The Kaplan–Meier survival analysis for local or combined events showed a significant difference between patients with residual disease detected by MRI with a mean EFS of 43 months (CI 95% 25 to 60) and patients without residual disease demonstrating a mean EFS of 81 months (CI 95% 72 to 90; *p* = 0.0014) (Figure 4). The hazard ratio for residual disease was 13.0 (CI 95% 2.3 to 37.7). The corresponding values in the HR group were 31 months (CI 95% 10 to 53) vs. 62 months (CI 95% 49 to 75; *p* = 0.015).

## 4. Discussion

The main objective of our study was to determine whether a relationship exists between residual tumors detected on MRI and local tumor control. The results indicate this association of residual disease with local or combined events. Thus, our finding might implicate changes in therapeutic strategies after surgery, especially in cases of residual disease and differences between a surgical report and an MRI.

CME, classified by the surgical report, can be seen as the standard of care, as it has been shown to improve local control and survival [23]. In 30/41 of our cohort, CME was reported. However, in 16/41 cases, postoperative MRI detected tissue suspicious of residual disease. The overlap between CME/IME and RD +/− on MRI was approximately 78%. This apparent discrepancy between the surgical report and MRI might be unexpected. However, surgical CME is defined as the removal of the entire visible or palpable tumor. Therefore, reaching certain regions sufficiently surgically may not be possible (e.g., along the distal mesenteric sections or retrocrural located lymph nodes). The lack of resection of clinically non-suspicious lymph nodes, from which a recurrence then arises, is also noteworthy in this context. Another reason for the discrepancy between the surgical report and the results of the MRI could be that only some of the patients received early postoperative imaging. Therefore, recurrence (in the case of CME) and tumor regression (in the case of IME) are theoretically possible. Furthermore, the median tumor volume of 2.2 mL in our observation was less than 5 mL. However, this volume is the minimal volume estimated by the surgeon, which is usually considered relevant for the indication of boost irradiation (e.g., SIOPEN protocol: HR-NBL-2; EudraCT: 2019-001068-31). Therefore, the results of the operations are at least equal to the international standard. The result, however, supports the efforts to harmonize the definitions, including surgical reporting, as recently published [27]. The value of the mentioned 5 mL residual tumor volume seems quite high. Following the International Neuroblastoma Response Criteria, a threshold of 1 cm is considered a complete response after resection [26]. Due to the possible inaccuracies of one-dimensional measurement comparing volume measurements used in this study, the lower limit of 1 cm is questionable. However, there is no evidence concerning the relevance of minimal tumor volume. Unfortunately, there is a lack of precise definitions for the residual tumor on imaging (MRI and CT). Regardless of tumor size, we defined residual tumor as conspicuous tissue with typical MRI characteristics for neuroblastoma on postoperative imaging, which was located at the same site and with comparable signaling on the preoperative imaging. In summary, the differences between the surgeon’s assessment and that of the findings on MRI are inevitable.

Our results may have significance for radiation planning. Current guidelines and protocols from COG and SIOPEN recommend that high-risk patients receive radiation therapy in the region of the tumor [9,28]. However, the long-term effects of radiation therapy have to be thoroughly considered despite its short-term benefits. The use of ionizing radiation, whether for diagnostic or therapeutic purposes, has been shown to increase the risk of developing secondary malignancies later in life [28,29,30,31]. It has also been shown that irradiation is related to musculoskeletal diseases, such as scoliosis [24]. Concerning the survival risk and the amount of resection, the SIOPEN protocol (HR-NBL-2; EudraCT: 2019-001068-31) also recommends radiation therapy of the tumor bed [28]. However, this strategy is controversial as recent data suggest that the impact of boost radiation on EFS is limited [19]. Previously, reduced radiation dose achieved local control rates similar to standard dose irradiation in high-risk NB [32]. The HR SIOPEN protocol also addresses the optimal treatment of residual disease by randomizing high-risk patients with macroscopic residual tumors to boost irradiation. Furthermore, it is currently discussed within the SIOPEN study group if further reduction or omission of radiotherapy after CME should be considered. In addition to these general considerations, a recently published study has shown how high the interobserver variability is in determining the operating plan [33]. Over time, radiotherapy techniques have advanced considerably, introducing image-guided, high-precision irradiation, and proton beam therapy to the field of neuroblastomas [34,35,36]. Therefore, exact three-dimensional tumor detection could be increasingly relevant for high-precision irradiations of neuroblastomas as it had already occurred in CNS disease [37,38]. As an outlook, new chances for monitoring radioligand therapies using hybrid methods (for example, with PET/MRI) could arise [39,40]. Therefore, clear definitions of residual tumor should be established in cases of overlap between MRI and resection results and in cases of potentially positive postoperative MRI and CME.

Our MRI study demonstrates a significant difference in local control between patients with and without residual tumors. There was an 88% and 100% (total cohort and HR group) improvement in mean local EFS. The results of this study can be discussed in two directions. On the one hand, it shows that an optimal surgical procedure minimizes the likelihood of local recurrence. Only one of nine patients with progression had no tumor remnants on MRI. On the other hand, the results underline the importance of the correct classification of even small residues by MRI. Thus, in five of eight patients with residual tumor and subsequent progression, the volume was less than 2 mL. This result implies to what extent such findings have been considered in studies to date. In addition, such tiny findings are not necessarily surgically detectable. However, neglecting small residual tumors may be why the differences in outcome between CME and IME are relatively small in the retrospective study by Holmes et al. [23]. On the other hand, the treatment of micrometastases is not the goal of surgical resection. In our evaluation, the status of IME, according to the surgical report, was not predictive of local recurrence. This is probably due to the low number of cases. However, the number of preoperative IDRFs showed a statistically significant correlation with progression, at least for the entire cohort. The result is in concordance with recently published data [41]. All other factors examined (N-MYC amplification status, histological regression, preoperative tumor volume) were not different. Due to the small number and the very uneven distribution (e.g., most tumors showed a regression level of 4), a combined multivariate analysis was not reasonably possible.

As mentioned above, timely imaging is necessary to assess the presence and extent of residual disease. However, no data are available regarding the ideal time point of postoperative imaging in neuroblastic tumors. The role of early postoperative MRI has been well investigated in the area of brain tumors [42,43,44,45]. Similar concepts might also be applied to neuroblastoma. In particular, the decision between residual tumor, recurrence, and unspecific tissue can only be made with certainty in this way. In our cohort, postoperative control was at a median of 92 days, so that tumor growth could have occurred. Although intraoperative imaging might be the ideal solution for assessing residual disease, we admit that this procedure is related to very complicated and complex logistical challenges. Additionally, current data need to be more comprehensive regarding the reliability of intraoperative imaging results in neuroblastoma. Despite these issues, postoperative imaging can be performed within 1 to 3 weeks after surgery (e.g., according to the SIOPEN protocol: HR-NBL-2; EudraCT: 2019-001068-31) after removal of drainages and discharge from intensive care to facilitate logistical issues.

Appropriate assessment of residual disease with quantitative and reliable measurements is necessary for the accurate allocation of patients. This is especially relevant as, currently, the surgical report (without reliable quantitative measurement) is handled as a reference regarding the resection status. Three different imaging methods can be applied for postoperative staging: MR, CT, or MIBG. Apart from the lack of radiation exposure, arguments favoring MRI are related to improved soft-tissue contrast and the application of DWI [46,47,48]. The value of DWI for assessing the degree of histological maturation has been described many times in recent years [46,47,48]. In our cohort inclusion from 2009, complete DWI data sets were not available for all patients. Therefore, we could not perform a quantitative analysis. However, the additional consideration and assessment of DWI facilitates the detection and characterization of small residuals in particular. On the other hand, there have been concerns about MRI accuracy for preoperative staging and surgical planning in the past, e.g., due to calcifications [49]. Despite this issue, MRI volume measurements in neuroblastic tumors have been shown to have high accuracy in comparison to pathological evaluation [25,50,51]. Additionally, three-dimensional volume sets, as shown in Figure 2, can be used as a basis for therapy planning of irradiation. We propose early postoperative MRI and a standardized follow-up examination protocol based on our findings. Furthermore, functional measurement methods should be included besides the volumetry of residual tumors, as suggested [52]. This would provide the instruments to sufficiently confirm the status of CME by imaging.

MIBG scintigraphy acquisition is currently a standard imaging procedure [4]. The disadvantages of planar scintigraphy are well known. Thus, SPECT or, at best, the hybrid method SPECT/CT is preferred here. However, the availability of SPECT/CT, in particular, is low. Although three-dimensional acquisition, including appropriate morphological resolution, is possible using SPECT/CT, radiation exposure of more than five mSv remains a limiting factor for repetitive scans. Current recommendations propose to perform ^18^F-PET/CT in MIBG-negative tumors. This functional imaging modality is particularly interesting for postoperative remission assessment, as PET/CT combines morphological resolution, including a good depiction of vessels with metabolic information of tumor tissue that might be used for outcome prediction [4]. PET/MRI combines both advantages with low radiation exposure [53]. In particular, the possibility of using an MIBG analog in PET (^18^F-MFBG) would be a revolution in the imaging of neuroblastoma [54].

As mentioned above, one limitation of our study is that we could not evaluate early postoperative MRI in all patients. Therefore, possible tumor growth cannot be completely ruled out. Furthermore, only about one-third of the patients who received a resection were included. The primary exclusion was because CT was often performed pre- or postoperatively instead of MRI due to better availability. Thus, we would not suspect a clear selection bias. Patients with different risk groups were included, with more than 50% of the patients in the HR group. Although this is the largest published group in which MRI has confirmed macroscopic residual tumor, our results require confirmation in a prospective trial.

## 5. Conclusions

In conclusion, the results indicate an association between residual disease detected by MRI and tumor progression events. Assessments by MRI demonstrate a possibility for objective and reliable quantification of residual disease after resection. We recommend volume measurement of the macroscopic residual disease. Particularly in small lesions, MRI as an independent method might be beneficial to surgical reports regarding the resection status. To correctly assess the problem of early tumor progression, an MRI should be performed as soon as possible after the operation.

## Figures and Tables

**Table 1:** Patient characteristics (n = 41).

Characteristics	Values
Number of patients (female)	41 (20)
Median age (months)	39 (24–71)
Neuroblastoma (N)	23
Ganglioneuroblastoma (N)	15
Ganglioneuroma (N)	3
High risk (N)	22
Intermediate risk (N)	8
Low risk (N)	11
N-MYC Amplification	
Positive (N)	7
Negative (N)	34
IDRFs	
Negative (N)	5
Positive (N)	36
CME (N)	30
Histological regression	
1	0
2	2
3	2
4	37
Median Time Span between MRI and Surgery	
Preoperative (days)	12 (5–30)
Postoperative (days)	95 (45–141)
Median preoperative tumor volume (mL)	28.1 (8.5–76.6)
Median postoperative tumor volume (mL)	2.2 (0.9–6.4)
In 16 patients	

Note: IDRFs = image-defined risk factors; CME = complete macroscopic resection; numbers in parenthesis are interquartile range.

**Table 2:** Characteristics all patients with event (N = 11).

Sex	Age at OP (Months)	N-MYC	Stage (INSS)	Risk	IDRFs (Numbers)	IME/CME	RD in MRI	Localization	Volume Preop (mL)	Volume Postop (mL)	Histology	Percentage of Resection	EFS (Months)	Event Localization
f	29	-	III	i	3	IME	+	PT + LN	92.4	15.9	NB	82.8	16	L *
m	75	-	IV	h	2	IME	+	PT	23.7	7.4	NB	68.8	14	L + M
m	65	-	III	i	4	IME	+	PT	18.5	0.3	NB	98.4	32	L
m	86	-	IV	h	1	IME	-		22.4		NB	100.0	16	M *
f	51	-	IV	h	2	CME	-		22.5		NB	100.0	20	M *
f	59	-	IV	h	1	CME	+	LN	15.1	0.2	NB	98.7	13	L + M
f	9	+	III	h	2	CME	+	PT	1109.8	1.2	NB	99.9	15	L + M
m	31	+	IV	h	2	CME	-		2.9		NB	100.0	24	L + M *
f	126	-	IV	h	2	CME	+	LN	293.2	1.8	GNB	99.4	12	L *
m	25	-	IV	h	4	CME	+	LN	31.6	3.6	GNB	88.6	37	L + M *
m	5	-	III	l	4	CME	+	LN	8.3	0.9	GNB	89.7	8	L + M

Note: INSS = International Neuroblastoma Staging System; CME = complete macroscopic; IME = incomplete macroscopic excision; IDRFs = image-defined risk factors; RD = residual disease; f = female; m = male; − negative; + positive; l = low; i = intermediate; h = high; LN = lymph node; PT = primary tumor; NB = neuroblastoma; GNB = ganglioneuroblastoma; GN = ganglioneuroma; L + M = local and metastatic; L = local; * = histological confirmed relapse.

**Table 3:** Analysis of risk factors.

	Local or Combined Event Total Cohort (N = 41)		Local or Combined Event HR Group (N = 22)			
	No (N = 32)	Yes (N = 9)	*p*	No (N= 16)	Yes (N =6)	*p*
Number of IDRFs	1 (1–2.75)	2 (2–4)	0.04	2 (1–3)	2 (1.75–2.75)	>0.05
N-MYC (N) −|+	27|5	7|2	>0.05	11|5	4|2	>0.05
Preoperative Tumor Volume (mL)	35.3 (7.5–73.2)	23.7 (11.7–192.8)	>0.05	35.3 (11.0–60.2)	27.7 (12.1–497.4)	>0.05
CME|IME (N)	24|8	6|3	>0.05	12|4	5|1	>0.05
Histological regression (N) 1|2|3|4	0|2|2|28	0|0|0|9	>0.05	0|1|2|13	0|0|0|6	>0.05
RD by MRI (N) −|+	24|8	1|8	0.0004	13|3	1|5	0.005
Postoperative Tumor Volume (mL) RD by MRI −|+	0|2.7 (0)|(1.2–7.1)	0|1.5 (0)|(0.4–6.4)	>0.05	0|1.4 (0)|(0.5–8.4)	0|1.8 (0)|(0.7–5.5)	>0.05

Note: IDRFs = image-defined risk factors; CME = complete macroscopic resection; RD = residual disease; numbers in parenthesis are interquartile range.

## Data Availability

Data are contained within the article. Additional data are available on request from the corresponding author.
